# Development of a scale to gain insight into the experience of living with chronic heart failure: The UNAV-Experience of Living with Chronic Heart Failure Scale

**DOI:** 10.23938/ASSN.1071

**Published:** 2024-04-16

**Authors:** Maribel Saracíbar-Razquin, Amparo Zaragoza-Salcedo, Jesús Martín-Martín, José Luis Cobo-Sanchez, Santiago Pérez-García, Aurora Simón-Ricart, Pilar Ara-Lucea, Leticia Jimeno-San Martín, Montserrat Ducay-Eguillor, Noelia De La Torre-Lomas, Jesica Pérez-Herreros, Maddi Olano-Lizarraga

**Affiliations:** 1 Universidad de Navarra School of Nursing Department of Adult Nursing Care Pamplona Spain; 2 Universidad de Navarra Innovation for a Person-Centred Care Research Group (ICCP-UNAV) Pamplona Spain; 3 Navarra Institute for Health Research (IdiSNA) Pamplona Spain; 4 Nursing Quality, Training, Research, Development and Innovation Unit Hospital Universitario Marqués de Valdecilla Santander Spain; 5 Instituto de Investigación Valdecilla (IDIVAL) Santander Spain; 6 Universidad Católica de Ávila. School of Nursing Ávila Spain; 7 Clínica Mompía. Mompía Cantabria Spain; 8 University Hospital 12 de Octubre Nursing Research and Teaching Madrid Spain; 9 Care Research Group (InveCuid+12) Research Institute Hospital 12 de Octubre Madrid Spain; 10 Complutense University School of Nursing, Physiotherapy and Podiatry Madrid Spain; 11 Clínica Universidad de Navarra Cardiology Department Pamplona Spain; 12 University Hospital 12 de Octubre Cardiology Department Madrid Spain; 13 Hospital Universitario Marqués de Valdecilla Advanced Heart Failure and Heart Transplant Unit Santander Spain

**Keywords:** Heart failure, Scale, Life Experience, Patient-reported Outcomes, Insuficiencia cardíaca, Experiencias de Vida, Medición de Resultados Informados por el Paciente

## Abstract

**Background:**

To date, there are no tools for the nursing staff to gain systematic insight on the experience lived by patients with chronic heart failure. The objective of this study was to develop a scale for this purpose.

**Methods:**

The study was conducted between January 2018 and December 2020 in three Spanish hospitals. The process described by DeVellis was used for the development of the scale. The items were built based on a phenomenological study and a systematic review of the literature. Next, feedback from a panel of experts was obtained, the scale was administered to a sample of patients with chronic heart failure, and a cognitive interview and an observational study were conducted to create the final version of the scale.

**Results:**

The first version of the scale had in seven domains and 76 items. After its evaluation by a panel of experts, it was reduced to a second version with six domains and 55 items. Following the administration of Version 2 to 17 patients (58.8% male, mean age 59.53, 70.6% classified as NYHA functional class II), five items were modified and two eliminated. Thus, the third version of the UNAV-CHF Experience Scale was composed of six domains and 53 items.

**Conclusions:**

This study presents the development of the UNAV-experience of living with chronic heart failure scale. It is an original and novel instrument that allows systematically explore this experience. A larger-scale study is necessary to confirm the validity of our scale.

## RESUMEN

**Fundamento:**

Actualmente no existen herramientas para explorar de forma sistemática la experiencia de vivir con insuficiencia cardiaca crónica (ICC). El objetivo de este estudio es desarrollar una escala para conocer la experiencia de vivir con ICC.

**Métodos:**

El estudio se realizó entre enero de 2018 y diciembre de 2020 en tres hospitales españoles. Se aplicó la teoría de DeVellis para el desarrollo de una escala. Los ítems se formularon a partir de un estudio fenomenológico y de una revisión sistemática de la literatura. Tras la opinión del panel de expertos, se administró la escala a una muestra de pacientes con ICC y se generó la versión final de la escala tras realizar una entrevista cognitiva y un estudio observacional.

**Resultados:**

La versión 1 de la escala constaba de siete dominios y 76 ítems. Tras la evaluación por el panel de expertos, la versión 2 se diseñó con seis dominios y 55 ítems. Tras su aplicación a 17 pacientes (58,8% varones, edad media 59,53 años, 70,6% clasificados como clase funcional II de la NYHA) se modificaron cinco ítems y se eliminaron dos, resultando la versión 3 de la Escala de Experiencia UNAV-CHF con seis dominios y un total de 53 ítems.

**Conclusiones:**

Este estudio presenta el desarrollo de la Escala UNAV-Experiencia de vivir con insuficiencia cardiaca crónica, instrumento original y novedoso que permite explorar esta experiencia de forma sistemática. Es necesario un estudio a mayor escala para confirmar su validez.

## INTRODUCTION

Chronic heart failure (CHF) is a complex syndrome, causing signs and symptoms such as dyspnoea, oedema, and fatigue^1^. Although advances in treatment options over the last few decades have led to improvements in its prognosis^2^, CHF, with a prevalence of over 10% among individuals aged >70 years, continues to have significant negative impact in terms of mortality, morbidity, and health-related quality of life^3^^,^^4^.

CHF is characterized by a progressive deterioration of the health status. Consequently, patients require complex treatments and self-care behaviours^5^ with significant effect on their well-being and other areas of live^6^^,^^7^. In recent decades, there has been increasing interest by nurses on the experience of living with CHF, and a considerable number of qualitative studies and literature reviews have been published^8^^-^^11^. The literature reveals the critical impact of CHF on people’s lives and points out that the health care given to these patients mainly focuses on the management of the disease and prevention of complications, while neglecting essential aspects in their lives^11^^-^^13^. To date, no tool has been developed in a structured and systematic manner to help clinicians understand the experience of living with CHF^14^^,^^15^.

It is necessary to adopt a person-centred approach to care, and for this, clinicians must have the appropriate tools. Many of the currently available scales or questionnaires designed to understand people with CHF focus on quality of life^14^, aspects related to the symptoms and functional capacity^16^^-^^19^, or self-care^20^^-^^22^. New scales are needed to obtain patient-reported outcomes and adopt tailored approaches for each experience and situation^23^, respecting their perception as a unitary being and understanding CHF as an experience integrated in their lives^24^. Moreover, this information should be collected using a systematic structure that informs patients, professionals, and managers about the situation, as this would facilitate the provision of the tailored care required by CHF patients^25^.

The purpose of this study is to develop a scale to gain insight into the experience of living with CHF.

## METHODS

### Theoretical framework

For this research, we considered the *Model of interpersonal relationship between the nurse and the person/family cared for*, which promotes person-centred care^26^. This framework focuses on empowering individuals/families to realize their own possibilities and find a meaning in their health experiences and lives. It conceives a human being as body and soul, an undivided set of parts (unique and different from others) in a continuous state of growth and novelty. Health is both a value and a lived experience as viewed from the perspective of each person, and it refers to the well-being of each person and recognition of their potential. Illness is part of the health experience and can be a major cause of changes in a person^12^^,^^27^^-^^29^.

### Design

**Figure 1:**
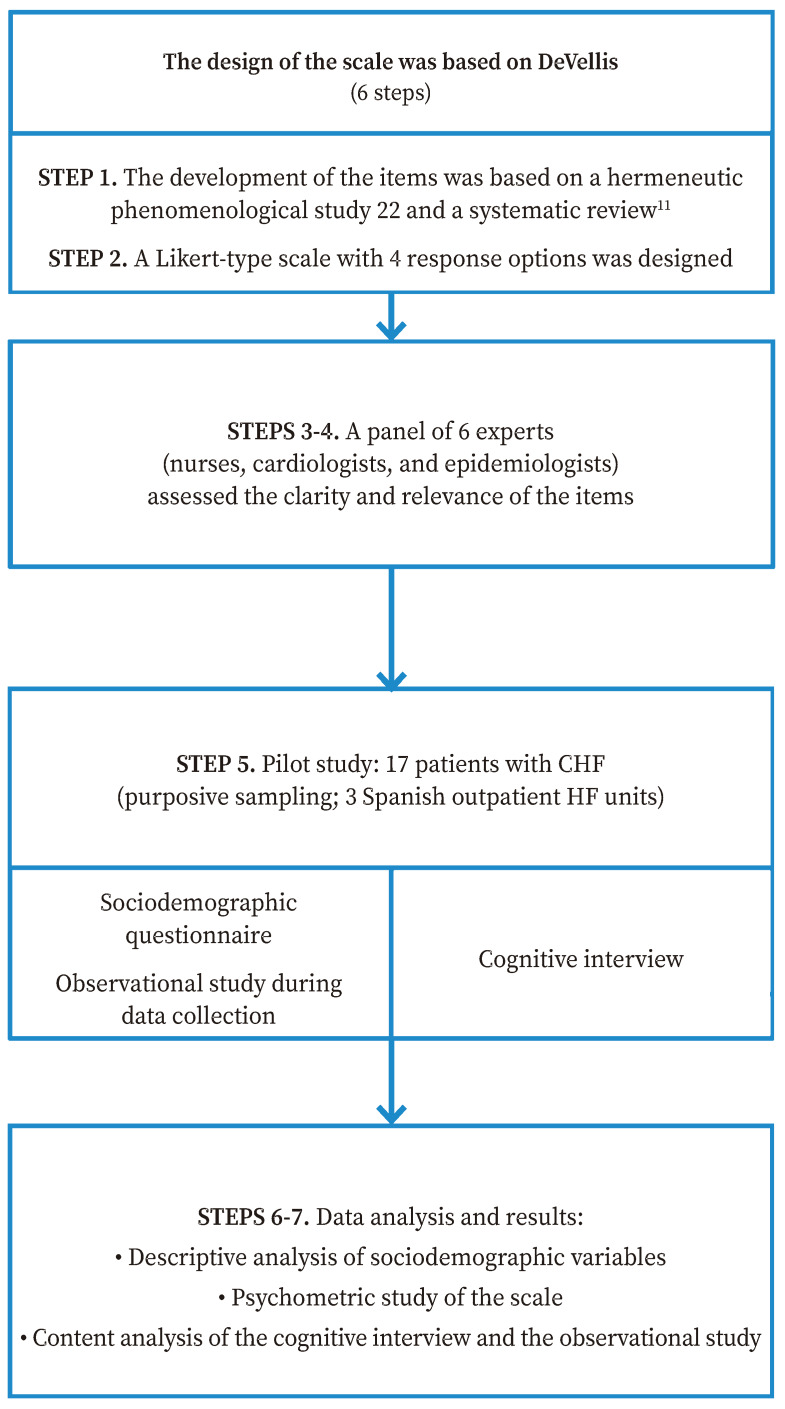
Scale design steps as described by DeVellis

The DeVellis-based theory of scale development was applied^30^ to design the UNAV-Experience of Living with Chronic Heart Failure Scale (UNAV-CHF experience scale). The main steps that were carried out were as follows: 1) generate a list of items; 2) determine the format of the instrument; 3) review content validity of Version 1 of the scale through a panel of experts; 4) analyse the feedback from the panel of experts and propose Version 2 of the scale; 5) apply Version 2 of the scale to a sample of patients (pilot study); 6) evaluate the items after the pilot test; and 7) optimize the instrument to generate Version 3 (Figure 1).

The initial list of items was based on a qualitative meta-synthesis^11^ and a hermeneutic phenomenological study^12^ about the meaning of the experience of living with CHF carried out by the research team, using the aforementioned theoretical framework (The *Model of interpersonal relationship between the nurse and the person/family cared for*)^26^.

A group of experts on scale design were contacted to maximize content validity: two CHF nurses, two cardiologists, and two epidemiologists. Independently, they completed two questionnaires: one on the relevance of each item for measuring the experience of living with CHF and another on whether each item was clear and concise, that is, if the syntax and semantics facilitated its understanding. The questionnaires was based on a 4-point Likert scale, where scores of 3/4 indicated a relevant and/or clear item and 1/2 that the item was irrelevant and/or unclear or diffuse; should the scoring be low, the expert had to explain why and suggest an alternative if she/he wanted to do so. The experts had 10 days to perform this assessment and return their responses.

Next, a cross-sectional study was carried out with a sample of patients with CHF as part of the scale development process as described below.

### Sample and recruitment

The study was carried out in the CHF outpatient units of three university hospitals from three Spanish regions (Navarra, Cantabria and Madrid). Seventeen patients were recruited using non-probability convenience sampling^31^ following DeVellis’ recommendation to include a minimum of 15 participants in pilot studies of scale design^30^. The selected participants had to meet the following criteria: 1) aged ≥18 years; 2) diagnosed with CHF with a functional class between II and IV according to the New York Heart Association Functional Class (NYHA-FC) classification; 3) had been diagnosed more than six months ago; 4) had not been hospitalized; 5) spoke and understood Spanish fluently; 6) had a normal level of consciousness with a state of health that allowed the patient to complete the questionnaires at that time; and 7) wanted to participate in the study and voluntarily signed an informed consent form.

Before starting the study, an informative session was held in each of the CHF units to which the nurses in charge of the follow-up of these patients were invited. These same nurses acted as gatekeepers, selecting participants based on the aforementioned criteria. When patients attended their scheduled consultation in the unit, the nurses informed them of the possibility of participating in the study and gave them the information sheet. For patients who agreed to participate, informed consent was explained to them, and the best time to collect data was scheduled. Five of the invited patients opted not to participate, claiming they did not want to stay longer than required to complete their medical examination for fear of contracting COVID-19.

### Data Collection

Data collection was carried out between June and September 2020 in one of the wards of the outpatient CHF units of the three hospitals. A single face-to-face meeting was held with each patient and two researchers from the team to collect the necessary data (13 to 45 minutes). During the meeting, two questionnaires were administered to the patient: a sociodemographic questionnaire and Version 2 of the Spanish UNAV-CHF Experience Scale. A Likert-type response scale was designed to measure response variability that included four response options ranging from *Strongly disagree* to *Strongly agree*. One of the researchers created these two questionnaires *ad hoc* using templates in the electronic database *Research Electronic Data Capture* (REDCap)^32^. The other researcher was a participant observer who recorded whether any item or term required clarification during the completion of the UNAV-CHF Experience Scale and any other observations regarding verbal and nonverbal communication that the patient manifested (for example, if she/he was annoyed or excited by any question). For this, a Microsoft Word *ad hoc* questionnaire was created in which the observer indicated whether the item was clear and/or added relevant observations based on the reaction of the patient when responding to each item.

Once this previous process was completed, a cognitive interview was held with each patient to collect his or her thoughts about aspects of the scale. Another *ad hoc* questionnaire was designed in REDCap^32^ and administered together with the sociodemographic questionnaire and the UNAV-CHF Experience Scale. This allowed us to gather and analyse the opinions of the participants regarding the relevance of the items, their experience when responding, the content, the suitability of the response options, the redundancy of the questions, and the length of the questionnaire. In addition, an open-ended question was included at the end, which allowed participants to suggest improvement ideas.

### Data Analysis

A descriptive analysis of the sociodemographic variables was performed; quantitative variables were presented as means and standard deviations (SD) and qualitative variables as frequencies, percentages, and ranges. Data were analysed using the SPSS 20.0 statistical software.

The results of the questionnaires administered in the non-participant observation study and the cognitive interview were analysed using the content analysis method. Specifically, this analysis was carried out following the method proposed by Burnard^33^. A repeated reading of the descriptions collected in the questionnaires was carried out by identifying the key words and descriptions collected as codes. Finally, these codes were grouped to produce findings that served to identify participants’ reactions and easiness to respond to the scale, as well as their opinions regarding the relevance of the items, the appropriateness of the scale, and their experience completing it.

### Ethical Considerations

Participants received oral and written information about the study at the CHF unit during their routine follow-up. They were ensured of their free participation, the confidentiality of the topics discussed, and the anonymization of the data. In addition, they were informed on their right to withdraw from the study at any time and ensured that nothing in this study would affect the care they were receiving at the hospital. All participants signed an informed consent form before any data was collected. In each of the participating hospitals, a researcher was assigned who was in charge of codifying the names of the study patients from his/her centre and kept the signed informed consent forms.

This research was approved by the Research Ethics Committee of the University of Navarra (reference number: 2017.125), the Clinical Research Ethics Committee of Cantabria (reference number: 2017.252), and the Ethics Committee of Drug Research of the University Hospital 12 de Octubre (reference number: 17/473).

## RESULTS

After a comprehensive analysis of the results of the qualitative meta-synthesis of the literature^11^ and the hermeneutic phenomenological study^12^ on the meaning of the experience of living with CHF carried out by the research team, the first version of the UNAV-CHF Experience Scale was built. It contained 76 items divided into seven domains.

The responses from the panel of experts on Version 1 of the UNAV-CHF Experience Scale were first analysed individually before comparing all the assessments, suggestions, and proposed modifications. If a score of 1 or 2 for the relevance of an item was assigned by more than 50% of the panel members, the item was omitted; if opposite scores were tied, the clarity and comments were assessed (Table 1).

**Table 1:** Domains and items in the preliminary versions of the UNAV-CHF Experience Scale

Domain	Version 1		Version 2			
	Id	Items	% of responses scored 3-4		Id	Items
			Relevance	Clarity		
Living with CHF involves profound personal changes	1	24	83.0	73.6	1	19
Having CHF can lead individuals to feel that they are no longer the same	2		56.7	66.6	Merged with Domain 1	
People living with CHF need to accept their situation	3	10	80.0	80.0	2	7
People with CHF need to feel that their lives are normal	4	11	83.3	81.8	3	9
People with CHF need to live with hope	5	10	86.6	88.3	4	8
People with CHF constantly think about death	6		90.5	92.9	5	6
People with CHF feel that their condition negatively impacts their close environment	7		90.7	87.0	6	7

UNAV-CHF Experience Scale: UNAV-Experience of Living with Chronic Heart Failure Scale; CHF: chronic heart failure. Relevance of the items: 3 = somewhat relevant, 4 = quite relevant. Clarity of the items: 3 = quite clear, 4 = very clear.

For the above process, several work sessions were held with the research group, which led to a second version of the UNAV-CHF Experience Scale with 55 items divided into six domains. Specifically, the items in domain 2, *Having CHF can lead individuals to feel that they are no longer the same*, were integrated into domain 1 and domain 2 was eliminated. The option *not applicable* was added to items 6 and 30, as they referred to people who worked outside their home. Likewise, an open-ended question was added at the end of the scale: *Would you like to add something else that has not been discussed and you think it might be important?*

### Development of the third version of the scale

#### Characteristics of the sample

Seventeen participants were included in the assessment of Version 2 of the UNAV-CHF Experience Scale. More than half of the sample were male and mean age of the participants was 59.53 years. Over half of the patients were married; 52.9% had completed secondary education/vocational training, and one third had only primary school education. With respect to employment, a large percentage of patients were retired (52,9%), unemployed (5,9%), or sick (11,8%). In terms of the level of disease involvement, almost three thirds of the patients were classified as NYHA functional class II and the remaining as class III. Finally, slightly more than half of the sample had low rates of comorbidity (Charlson Index between 1 and 2 points; only one tenth obtained scores >5 points). Mean number of years since diagnosis was 9.47 (SD: 6.15) (Table 2).

#### Results of the cognitive interviews

Patients reported that Version 2 of the UNAV-CHF Experience Scale was relevant for informing health professionals about their situation. Most (82.35%) thought that the scale was comprehensive. In addition, all participants assured that none of the questions made them feel uncomfortable when answering.

**Table 2:** Sociodemographic data of participants (17) for assessing the pilot scale (Version 2)

Variable	n (%)
*Sex*	
Female	7 (41.2)
Male	10 (58.8)
*Age (years)*	
Mean (SD)	59.53 (15.54)
Range	31-82
*Marital status*	
Single	3 (17.6)
Married	10 (58.8)
Divorced	3 (17.6)
Widowed	1 (5.9)
*Education level*	
Primary education	6 (35.3)
Secondary education/ vocational training	9 (52.9)
Higher education	2 (11.8)
*Employment*	
Full-time	1 (5.9)
Part-time	2 (11.8)
Works from home	2 (11.8)
Retired	9 (52.9)
Work leave	2 (11.8)
Unemployed	1 (5.9)
*Years since diagnosis*	
Mean (SD)	9.47 (6.15)
Range	1-25
*Functional class (NYHA)*	
II	12 (70.6)
III	5 (29.4)
*Charlson Comorbidity Index*	
1-2 points	10 (58.8)
3-4 points	5 (29.4)
>5 points	2 (11.8)

SD: standard deviation; NYHA: New York Heart Association Functional Class classification

In terms of the suitability of the response possibilities, 64.7% indicated that the options covered how they wanted to respond; however, 35.3% thought that they were unsuitable. In the latter group, three patients specified the need to readjust the number of options: one suggested an increase in the number of possible answers and the other suggested a decrease, and the other one pointed out the benefit of introducing a scale of only numerical responses. Most patients (82.35%) considered that the interview was long enough. Two patients made questions related to religion and/or family life that did not correspond to their situation and 70.58% of the patients made no suggestions for improvement.

#### Results of the observational study

The observational study confirmed that most Version 2 items of the UNAV-CHF Experience Scale were clear. However, patients from the three hospitals found difficult/annoying to answer items 24 and 42 (related to faith and religious beliefs) because it did not apply to them.

Items 6 (*This disease has affected my work*), 7 (*My illness has influenced my relationship with my partner*), and 31 (*Being able to work makes my life feel normal*) did not apply well, as some patients did not work or have a partner.

Several items required term clarification or on how to score the answer: items 12 (*The changes that have occurred in my habits make me feel bad*), 17 (*The changes my body has undergone make me not recognize myself*), 18 (*The changes in my state of mind make me not recognize myself*), 21 (*Despite the illness, I am able to continue enjoying life*), and 29 (*I can do the household chores that are important to me*).

#### Optimization of Version 2 to generate Version 3 of the UNAV-CHF Experience Scale

Version 2 of the instrument was optimized following step 7 of the DeVellis development proces ^30^. The research team shared the results of the various measurements and assessed their consistency with respect to the conceptual framework for the development of our scale. This resulted in the following changes: items 24 (*My religious beliefs help me accept what happens to me with serenity*) and 55 (*My family members have assumed some of responsibilities that I had*) were eliminated (redundant content); items 6, 7, and 31 incorporated *not applicable* as a response option; and the wording was modified in items 12 (*habit* was replaced by *routine* in *The changes that have occurred in my habits make me feel bad*) and 42 (*My faith gives me strength to move forward* was replaced with *My beliefs give me strength to move forward*).

**Figure 2:**
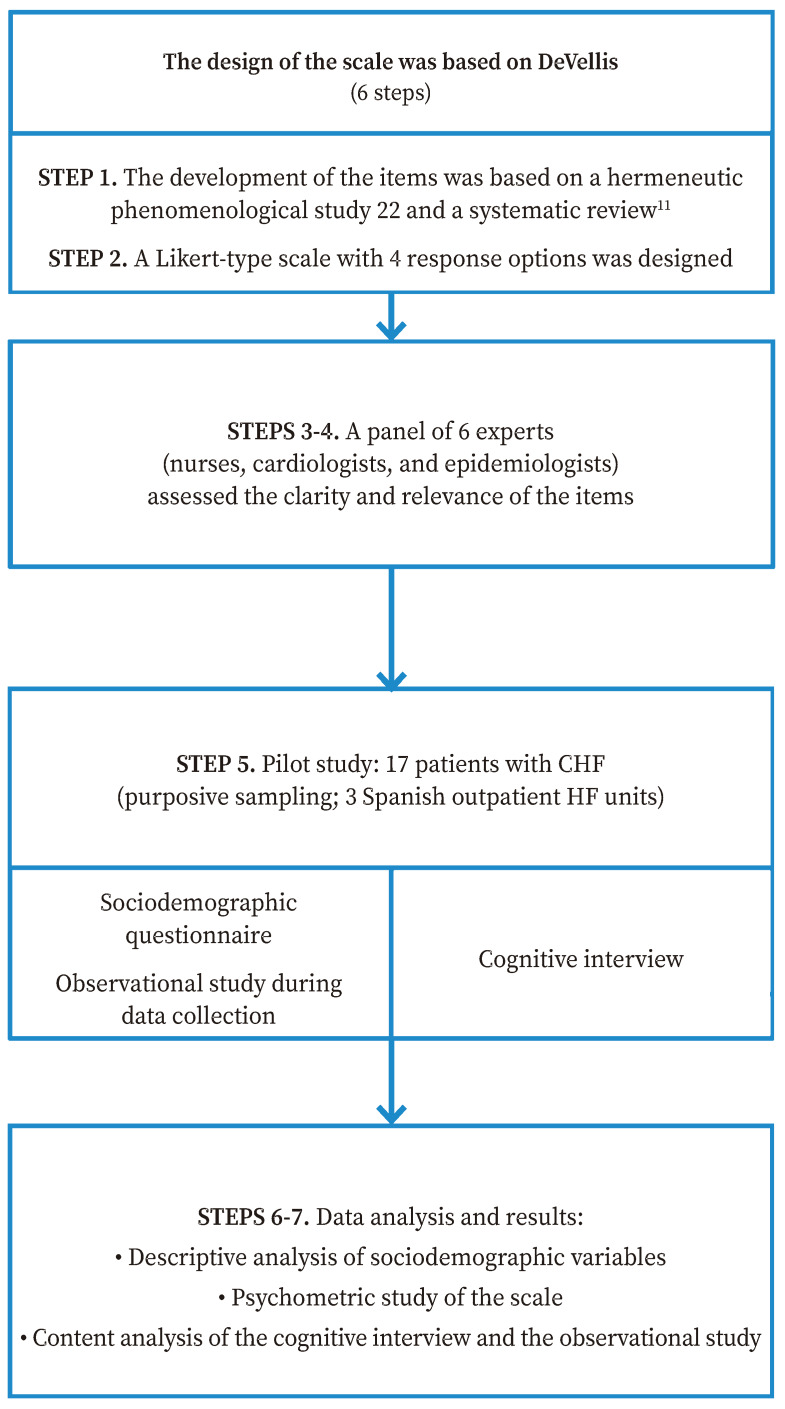
Structure of version 3 of the UNAV-CHF Experience Scale.

These changes lead to Version 3 of the UNAV-CHF Experience Scale, composed of 53 items divided into six domains and an open-ended question at the end (Figure 2, Table 3).

UNAV-CHF Experience Scale: UNAV-Experience of Living with Chronic Heart Failure Scale; CHF: chronic heart failure.

**Table 3:** Topics addressed in the domains and some of the items of the UNAV-Experience of Living with Chronic Heart Failure Scale

Item	Topics addressed in the domains/items
*Profound changes*	
5	Feeling of loneliness
11	Sense of usefulness
17	Body changes
*Acceptance*	
19	Acceptance of changes
20	Adapting to change
21	Enjoying life
*Normality*	
25	Perception of illness
27	Daily routine
28	Household tasks
*Hope*	
34	Overcoming difficulties
37	Motivation for living
38	Sense of calm
*Death*	
43	Feeling of fear
44	Anguish about dying
46	Death wishes
*Family*	
48	The suffering of the family
50	Feeling of burden
52	Hiding symptoms

## DISCUSSION

This study presents the process of developing the first scale aimed at understanding the experience of living with CHF. It is a novel and innovative contribution to this area of knowledge, as it will help nurses to assess CHF patients´ experience in a systematic and structured manner and promote a tailored care approach for this population.

The dimensions that compose this scale are derived from scientific evidence from previous research^11^^,^^12^^,^^27^^,^^28^. Moreover, the evidence has been supported by other authors^7^^,^^34^ who explored how people with CHF experience the symptoms, their emotional responses, and the impact CHF has on their daily lives and psychological spheres, as well as on their activities and social relationships. Other studies have previously identified the need to accept their situation and make sense of what is happening to them^35^^,^^36^. Another dimension included in the UNAV-CHF Experience Scale seeks to measure the presence of a perception of normality and of the aspects that help CHF patients perceive a normal life. This phenomenon has been previously studied in people living with other chronic diseases such as cancer^37^, chronic obstructive pulmonary disease^38^, diabetes^39^, and rheumatoid arthritis^40^, denoting the relevance of assessing these factors to determine whether a person is coping adequately with their disease. Other authors have studied another key issue assessed in our scale, i.e., the value of hope in the lives of people with CHF^41^^,^^42^. Evidence supports the need to know whether CHF patients have thoughts of death and the impact these thoughts have on their lives^43^^-^^45^. Finally, several studies explore the perception of discomfort experienced by this population when thinking that their situation negatively influences people in their immediate environment^46^.

The UNAV-CHF Experience Scale fills a gap identified in the literature^14^^,^^15^ that often prevents health professionals from talking to CHF patients about their life experiences and deepest concerns in a scientific manner and acknowledge the importance this experiences deserves^12^. To date, this knowledge is occasionally obtained during informal nurse-patient conversations or in the context of a research^8^^-^^11^^,^^47^, limiting its use to the care provided to CHF patients^12^. This new scale offers a valuable aid to initiate conversations about intimate issues while offering a structure to record, provide continuity, and intervene in many of the aspects that most concern people who live with CHF in day-to-day basis.

The introduction of this scale is a very important advancement when planning patient-tailored care. To enable this type of care, personal experiences, life history, family and environment support, strengths and weaknesses, and the objectives and desires of each person must be considered^23^. The UNAV-CHF Experience Scale may help gain further information on these aspects because the questions patients has to answer are related to his/her experience with CHD, the relationships with the immediate environment, the desires, concerns, and sources of personal strength, and the open-ended question at the end.

One of the main limitations of this study was the small sample size used to pilot the scale. In addition, we used purposive sampling, which limited the representativeness of the population.

The sample was heterogeneous as the participants were from three hospitals in different Spanish regions. Robustness of the theoretical framework that supported the content of the items based on scientific evidence verified by the authors is significant. All steps for the methodological design of a scale were rigorously followed and are described in detail throughout the text, which reflects the transparency and replicability of this study.

This study presents the first phase of the development process of the UNAV-CHF Experience Scale, an original and novel instrument that will allow the experience of living with CHF to be explored in a systematic and structured manner.
